# Factor analysis and evaluation of one-year test-retest reliability of the 33-item Childhood Trauma Questionnaire in Chinese adolescents

**DOI:** 10.3389/fpsyg.2024.1384807

**Published:** 2024-08-23

**Authors:** Jiamei Zhang, Zhipeng Wu, Min Chen, Yan Gao, Zhening Liu, Yicheng Long, Xudong Chen

**Affiliations:** ^1^Department of Psychiatry, The Second Xiangya Hospital, Central South University, Changsha, China; ^2^National Clinical Research Center for Mental Disorders, The Second Xiangya Hospital, Central South University, Changsha, China; ^3^School of Mental Health, Jining Medical University, Jining, China

**Keywords:** Childhood Trauma Questionnaire, childhood trauma, factor structure, test-retest reliability, childhood abuse

## Abstract

The 33-item Childhood Trauma Questionnaire (CTQ-33) is a recently developed tool expanded from the 28-item Childhood Trauma Questionnaire (CTQ-28) to assess childhood trauma events, which showed good test-retest reliability over 2 weeks. However, little is known regarding the factor structure and long-term test-retest reliability of the CTQ-33. To fill such a gap, this study investigated the factorial validity of the CTQ-33 and test-retest reliability of the scale over a relatively long interval of 1 year. Data on demographics, the CTQ-33 scores, and mental health statuses such as depressive/anxiety symptoms were collected in Chinese adolescents (*n* = 188) twice across a one-year period. Results of the confirmatory factor analysis (CFA) revealed that the Chinese version of CTQ-33 has close factor validity when compared to the original CTQ-28 in college students. Furthermore, the total and most subscale scores of the CTQ-33 have fair to good test-retest reliability (intra-class correlation coefficients >0.6 for the total score, and > 0.4 for most subscales), except for the physical abuse subscale. Moreover, we replicated previous findings of significant positive relationships between levels of different childhood trauma subtypes using the CTQ-33. These findings provide initial evidence supporting that the CTQ-33 is overall reliable to assess childhood traumatic events in adolescents over relatively long intervals.

## Introduction

It has consistently been shown that childhood trauma results in a host of psychological health impairments across the lifespan (Stanhope et al., 2022; LeMoult et al., 2020), which is associated with serious clinical symptoms of mental, such as depression, anxiety disorder, post-traumatic stress disorder, eating disorders, drug abuse, sexual dysfunction disorders, personality disorders and separation, insomnia and suicidal (LeMoult et al., 2020; Wei et al., 2022; Racine et al., 2021; Cabanis et al., 2021; Maercker et al., 2022; Spiegel et al., 2022). A study of children living in the United States found that 45% had experienced one or more forms of childhood trauma (Watters et al., 2023). Further, A multicenter, large-sample study indicated that about 80% of Chinese reported experiencing one or more kinds of childhood trauma events (Zhang T. et al., 2023). As childhood trauma is highly prevalent in adolescents, it has been attributed as one of the important factors in the progression of psychological disorders (Cuijpers et al., 2011). In recent years, research and practice on adverse childhood experiences have risen, while a more comprehensive understanding of them is still needed (Narayan et al., 2021). Therefore, better measurement of individual childhood trauma levels has positive significance for early active intervention of mental disorders.

In the field of childhood trauma research, the 28-item Childhood Trauma Questionnaire (CTQ-28) is one of the most commonly used scales, which includes five subscales to assess five different subtypes of traumatic events (abuses and neglects) during childhood (Burgermeister, 2007; Huang et al., 2021; Wu et al., 2022; Bernstein et al., 2003). Bernstein et al. (2003) have indicated that based on confirmatory factor analysis, the CTQ-28’s factor structure has good predictive ability in English-speaking American samples. The Chinese version of CTQ-28 has also been widely used in the Chinese population, which showed acceptable reliability, internal consistency, and construct validity among Chinese samples (He et al., 2019; Zhao et al., 2005).

Recently, many studies have shown that overprotection/overcontrol (OP/OC) experiences during childhood can also be considered as a kind of traumatic experience, which is a risk factor for various mental problems (Azar et al., 2007; Yoshida et al., 2005). Therefore, expanded from the conventional CTQ-28, Şar et al. (2021) developed a 33-item version of the Childhood Trauma Questionnaire (CTQ-33) with OP/OC as an additional subscale. Previous research has shown that there were significant positive correlations between different traumas (abuses, neglects, and OP/OC) in the CTQ-33, and there are both shared and unique associated factors between different trauma subtypes (Zhang J. et al., 2023). The CTQ-33 soon attracted the attention of researchers from multiple countries and has been translated into multiple languages including the English, Turkish, and Chinese versions (Wu et al., 2022).

The internal consistency and two-week test-retest reliability of the CTQ-33 have been tested in previous research, and the results showed good internal consistency and excellent test-retest reliability (Wu et al., 2022). However, little is known regarding the structural validity of the CTQ-33 and test-retest reliability of the scale over relatively long intervals. This is important because as a retrospective self-reported scale, the results of the CTQ-33 may be influenced by recall bias, which could lead to the deviation of measurement results (Xiang et al., 2021). Thus, before being used in large-scale clinical studies, assessing the long-term test-retest reliability and fit goodness of scale structure of the CTQ-33 is warranted.

To address the above concerns, the present study examined the factorial structure and test-retest reliability of the Chinese version of CTQ-33 over a relatively long interval (one year) in Chinese adolescents. The results are anticipated to provide initial evidence for the scale structure validity of CTQ-33 and the reliability of CTQ-33 in clinical studies when used for relatively long terms.

## Methods

This study included a total of 188 Chinese adolescents who were recruited from two universities in China (*n* = 58 from Central South University, and *n* = 130 from Jining Medical University). These participants were drawn from a larger, previously published cross-sectional survey conducted from September 2021 to October 2021 (Zhang J. et al., 2023). From September 2022 to October 2022 (one year after the baseline survey), participants who were willing to participate in this study were asked to complete the same scales as at the baseline. All students completed the survey online through a famous platform in China, “Questionnaire Star”.^1^ The questionnaire is issued by each student in charge. Participants have been excluded if they met the following criteria: (1) had a previous diagnosis of any psychiatric disease; (2) had missing data on any demographic or clinical variables. In addition, we have also excluded those with a response time of less than 7 minutes during any wave of the survey to ensure the quality of the survey results, as done in other studies (Sun et al., 2023). The final included 188 participants are consisted of 125 females and 63 males, with an average age of 18.40 ± 0.95 years (Table 1).

**Table 1:** Demographic and clinical characteristics of the participants (*n* = 188).

	Range (Baseline)	Range (Follow-up)	At baseline	At follow-up	Paired *t*-test comparisons
			Mean ± SD	Mean ± SD	
Age	16–23	–	18.40 ± 0.95	–	–
Gender (female/male)	–	–	125/63	–	–
Nationality (Han/ minority)	–	–	177/11	–	–
PHQ-9 score	0–27	0–22	4.33 ± 3.68	4.23 ± 4.48	*t* = 0.340, *p* = 0.734
GAD-7 score	0–19	0–21	3.20 ± 3.12	3.45 ± 4.03	*t* = −0.862, *p* = 0.390
**CTQ-33 scores**					
Emotional abuse	5–21	5–14	6.27 ± 2.13	6.21 ± 1.89	*t* = 0.393, *p* = 0.695
Physical abuse	5–15	5–10	5.28 ± 1.11	5.27 ± 0.94	*t* = 0.162, *p* = 0.871
Sexual abuse	5–20	5–20	5.29 ± 1.48	5.16 ± 1.19	*t* = 1.899, *p* = 0.059
Physical neglect	5–17	5–15	7.40 ± 2.46	7.36 ± 2.52	*t* = 0.192, *p* = 0.848
Emotional neglect	5–21	5–25	8.07 ± 3.11	8.56 ± 3.93	*t* = −1.930, *p* = 0.055
Overprotection/Overcontrol	5–19	5–17	8.41 ± 3.32	8.05 ± 2.92	*t* = 1.847, *p* = 0.066
Total	30–93	30–87	40.72 ± 9.26	40.61 ± 9.24	*t* = 0.181, *p* = 0.857

CTQ-33, the 33-item Childhood Trauma Questionnaire; GAD-7, the 7-item Generalized Anxiety Disorder Scale; PHQ-9, the 9-item Patient Health Questionnaire.

All participants completed the CTQ-33 (Chinese version) in both the baseline and follow-up surveys. The CTQ-33 is a 33-item retrospective self-reporting measure that includes six subscales: emotional neglect (EN), emotional abuse (EA), physical neglect (PN), physical abuse (PA), sexual abuse (SA), and OP/OC (Şar et al., 2021). Each subscale question contains five items and is answered on the 5-point Likert scale (1 = “never true” to 5 = “very often true”). The five new items (OP/OC) of the CTQ-33 were previously translated by our research team (Wu et al., 2022). Translations were performed by the corresponding author of this article, Dr. Yicheng Long. Chinese versions of the five OP/OC items were further translated back into English independently by another member of our team, Dr. Danqing Huang. Another clinician, Mr. Zhibiao Xiang, compared the original and translated entries to verify the consistency. The Chinese version of the CTQ-33 was shown to have comparatively good test-retest reliability in a two-week short term (Wu et al., 2022). Except for the CTQ-33, the following clinical assessments were also performed at both baseline and follow-up to investigate the possible relationships between childhood trauma and mental health statuses: the 9-item Patient Health Questionnaire (PHQ-9), and the 7-item Generalized Anxiety Disorder Scale (GAD-7). The PHQ-9 (Kroenke et al., 2001) is a self-reported questionnaire to assess the severity of depressive symptoms over the past 2 weeks. The Chinese version of PHQ-9 has been indicated good internal reliability (Wang et al., 2014). The total score of PHQ-9 ranges from 0 to 27. Participants scoring higher on the PHQ-9 were considered to have more severe depression symptoms. The GAD-7 (Spitzer et al., 2006) is a 7-item self-report questionnaire to assess the symptoms of anxiety. Responses to each item range from 0 to 3 with total scores of the GAD-7 ranging from 0 to 21; higher scores reflected higher anxiety levels. The Chinese version of GAD-7 has been shown to have good reliability and validity in samples of Chinese (Tong et al., 2016; Zhang et al., 2021).

Paired *t*-tests were used to test possible differences in scores of all scales (the CTQ-33, the PHQ-9, and the GAD-7) between the baseline and follow-up. Confirmatory factor analysis was carried out using the program AMOS 24 to test the original factor model (Bernstein et al., 2003). CFA results were reported with χ2/df, the root mean square error of approximation (RMSEA), the comparative fit index (CFI), and the Tucker-Lewis index (TLI). When the χ^2^/df, value of ≤3; and RMSEA <0.1, The CFI and TLI values >0.90 indicate adequate good fit, while values between 0.80 and 0.90, χ^2^/df value of <5, indicate that the structure is suitable for a good fit (Hu and Bentler, 1998, 1999). The one-year test-retest reliability of the CTQ-33 (including the total score and subscale scores) was assessed by the two-way random effects intra-class correlation coefficients (ICCs). According to ICC values, the reliabilities of scores can be classified into different levels: ICC < 0.4 indicates poor reliability; 0.4 ≤ ICC < 0.6 indicates moderate reliability; 0.6 ≤ ICC < 0.75 indicates good reliability; and ICC ≥ 0.75 suggests excellent reliability (Long et al., 2023). The *p* values were corrected across the 7 factors using the Benjamini–Hochberg false discovery rate (FDR) corrections, and a corrected *p*-value of <0.05 was considered significant.

## Results

There were no significant differences in total score and scores of each subscale in the CTQ-33 between baseline and follow-up data (all *p* > 0.05, Table 1). Furthermore, both mean depression scores (the PHQ-9 scores) and mean anxiety scores (the GAD-7 scores) did not change significantly over the one-year period (both *p* > 0.05, Table 1).

The confirmatory factor analysis was presented in Table 2. In the CTQ-33 of baseline data, the χ^2^/df value was calculated as 2.610 (*p* < 0.001), with RMSEA = 0.093, CFI = 0.780, and TLI = 0.754. In the CTQ-33 of follow-up data, the χ^2^/df value was calculated as 2.414 (*p* < 0.001), with RMSEA = 0.087, CFI = 0.825 and TLI = 0.805. For comparisons, we also performed the same analysis for the original version of CTQ-28 by excluding the five additional items in the CTQ-33. Among the CTQ-28 of baseline data, the χ^2^/df value was calculated as 3.080 (*p* < 0.001), RMSEA 0.105, CFI 0.778, and TLI 0.748; and in the CTQ-28 of the follow-up data, the χ^2^/df value was 2.520 (*p* < 0.001), with RMSEA = 0.090, CFI = 0.854 and TLI = 0.835.

**Table 2:** Fit indices of the confirmatory factor analyses.

Group	χ^2^	*p*-value	χ^2^/df	TLI	CFI	RMSEA
CTQ-33 (baseline)	1018.005	<0.001	2.610	0.754	0.780	0.093
CTQ-33 (follow-up)	941.314	<0.001	2.414	0.805	0.825	0.087
CTQ-28 (baseline)	816.120	<0.001	3.080	0.748	0.778	0.105
CTQ-28 (follow-up)	667.800	<0.001	2.520	0.835	0.854	0.090

CFI, the comparative fit index; CTQ-28, the 28-item Childhood Trauma Questionnaire; CTQ-33, the 33-item Childhood Trauma Questionnaire; RMSEA, the root mean square error of approximation; TLI, the Tucker-Lewis index.

All scales showed good internal consistencies in the surveyed participants (Cronbach’s α coefficients = 0.743 for the CTQ-33, 0.842 for the PHQ-9, and 0.862 for the GAD-7; estimated based on baseline data. Cronbach’s α coefficients = 0.712 for the CTQ-33, 0.888 for the PHQ-9, and 0.923 for the GAD-7; estimated based on follow-up data). The CTQ-33 showed a good one-year test-retest reliability (ICC = 0.619) when estimated as a whole. Furthermore, most subscales of the CTQ-33 showed moderate (EA and PN, 0.4 ≤ ICC < 0.6), good (OP/OC and EN, 0.6 ≤ ICC < 0.75), or excellent (SA, ICC > 0.75) test-retest reliabilities except the PA subscale (ICC < 0.4) (Table 3).

**Table 3:** One-year test-retest reliabilities (ICCs) of the CTQ-33.

CTQ-33 scores	ICC (with 95% confidence intervals)
Emotional abuse	0.487* (0.370, 0.589)
Physical abuse	0.136* (−0.007,0.274)
Sexual abuse	0.763* (0.697,0.817)
Physical neglect	0.429* (0.304, 0.538)
Emotional neglect	0.630* (0.536, 0.709)
Overprotection/Overcontrol	0.642* (0.550, 0.719)
Total	0.619* (0.522, 0.700)

*Significance at the 0.05 level; CTQ-33, the 33-item Childhood Trauma Questionnaire; ICC, intra-class correlation coefficients.

As shown in Table 4, significant positive correlations were found between all pairs of subscales in the CTQ-33 (*p* < 0.05).

**Table 4:** Pearson correlation coefficients between different subscale scores of the CTQ-33.

	Physical abuse	Emotional neglect	Emotional abuse	Physical neglect	Sexual abuse	Overprotection/overcontrol
Emotional neglect	0.237^*^					
Emotional abuse	0.420^**^	0.346^**^				
Physical neglect	0.288^**^	0.441^**^	0.330^**^			
Sexual abuse	0.420^**^	0.197^*^	0.499^**^	0.306^**^		
Overprotection/overcontrol	0.250^*^	0.387^**^	0.367^**^	0.322^**^	0.232^*^	
Total	0.529^**^	0.731^**^	0.695^**^	0.688^**^	0.555^**^	0.725^**^

The correlation coefficients shown here were calculated based on baseline data.*p < 0.01; **p < 0.001.

## Discussion

To the best of our knowledge, this is the first effort to analyze the factor structure of the CTQ-33 and evaluate the long-term reliability of the CTQ-33. Overall, the results showed that the Chinese version of CTQ-33 has close factor validity when compared to the original CTQ-28, and the total score and most subscale scores of the CTQ-33 have fair to good test-retest reliability.

Previous research examining the Chinese version of the CTQ-28 has indicated relatively satisfactory construct validity (CFI = 0.80/RMSEA = 0.06) for the CTQ-28 among Chinese populations (Zhao et al., 2005). In the present study, the results of the CFA showed very close CFI values (around 0.80 for both the CTQ-28/CTQ-33 and for both the baseline/follow-up data, see Table 2). Importantly, our results showed that at both baseline and follow-up, the six-factor structure of CTQ-33 has close and even slightly better (as indicated by lower RMSEA and higher CFI/TLI values, e.g., CFI = 0.778 for the CTQ-28 and 0.780 for the CTQ-33 at baseline) factor validity when compared to the five-factor structure of CTQ-28 in Chinese college student sample (Table 2). Therefore, we suggested that the CTQ-33 could have at least a close factor validity compared to the original CTQ-28 in Chinese samples. Nevertheless, it should be noted that such a construct validity is still lower than that of the English-version CTQ-28 as observed in the study by Bernstein et al. (2003) (CFI > 0.90; RMSEA <= 0.06 in four different groups). Thus, we propose that further improving the construct validity of the CTQ-28/CTQ-33 in Chinese could be considered as a priority in future studies on this scale.

Our results also proved that the CTQ-33, expanded from the original CTQ-28, had overall good reliability for Chinese university students in terms of internal consistency (Cronbach’s α coefficients = 0.743 in baseline and 0.712 in follow-up data, both >0.7) and test-retest reliabilities when retested over a one-year interval (ICC = 0.619 for the total score, and ICCs >0.4 for most subscales, Table 3). Previous studies have reported good test-retest reliabilities of the CTQ-28 when retested over both short and long terms. For instance, an earlier study reported that the CTQ-28 showed excellent two-week test-retest reliability (ICC > 0.8 for the total score) in two hundred schizophrenia patients (Jiang et al., 2018). In another study, it was shown that the CTQ-28 had good long-term test-retest reliability when re-evaluated over periods ranging from 2 to 41 months (Xiang et al., 2021). As for the CTQ-33, Wu et al. (2022) have validated the CTQ-33 in 248 young healthy participants in a previous study, which showed the Chinese version of CTQ-33 had a good internal consistency (Cronbach’s α coefficient = 0.733) and excellent test-retest reliability over a short period (two-weeks: ICC = 0.861). However, as far as we know, no prior studies have explored the long-term test-retest reliability of the CTQ-33. Here, the present study may fill such a gap for the first time and offer important evidence for the reliability of this relatively new measurement in mental health studies.

In the present study, most subscales of the CTQ-33 showed at least moderated test-retest reliabilities (ICCs >0.4, Table 3). In six subscales in the CTQ-33, the SA had the highest long-term test-retest reliability, which is consistent with the prior research (Xiang et al., 2021; Wielaard et al., 2018). Furthermore, it is noteworthy that the additional (compared to the CTQ-28) OP/OC subscale had the second highest reliability (ICC = 0.642). Such results may further support that the newly added items in the CTQ-33 are as promising as the original ones. On the other hand, the PA subscale showed a relatively low test-retest reliability. As discussed in some previous studies with similar results, this may be related to differences in the definitions of physical abuse/neglect across different cultures (Xiang et al., 2021; Wielaard et al., 2018). For example, Chinese people might agree with the adage “spare the rod and spoil the child,” while people from other countries may not agree (Wu et al., 2022). Moreover, the protective effect of a Chinese traditional cultural factor, namely filial piety, might help diminish an individual’s understanding of childhood abuse (Ng et al., 2011). Nevertheless, our results still support that the total score and most subscales of the CTQ-33 are reliable, especially for the new OP/OC subscale.

Highly consistent with the previous studies (Wu et al., 2022; Şar et al., 2021), there were significant positive correlations between different subscale scores in the CTQ-33, including the OP/OC subscale (Table 4). As discussed earlier (Wu et al., 2022), such results reinforce the opinion that OP/OC is a subtype of childhood traumatic experience, and that the OP/OC subscale does not extensively deviate from the original CTQ-28. In particular, our results show that OP/OC is positively correlated with sexual abuse. Although no studies have been conducted to clearly report such a phenomenon, previous studies on people with social anxiety disorder found that participants experienced higher levels of traumatic experiences such as sexual trauma and parental overcontrol (Norton and Abbott, 2017). In addition, another research has found that people with borderline personality disorder suffer more sexual and physical abuse and more overprotective fathers during childhood (Byrne et al., 1990). What’s more, there are case reports of the patient suffering from both excessive control over by her mother and trauma such as sexual assault by her uncle (Adachi et al., 2024). Therefore, we speculate that OP/OC is a factor of poorer family functioning which is associated with sexual abuse.

There are some limitations in the present study and potential future directions to be noted. Firstly, we cannot draw firm conclusions about cause and effect due to the cross-sectional survey, therefore, further longitudinal studies are needed to address this limitation. Secondly, the current study used a self-reported retrospective scale, which may have led to memory-related biases. What’s more, as the overall number of questions in the study was large, although we had excluded those with shorter filling times (less than 7 min), it cannot be completely ruled out that some subjects were not careful enough when filling in the data, or lacked interaction with participants’ understanding of the questions, or they may consciously or unconsciously modify their responses leading to an invalid assessment, for example, the individual with alcoholism would affect their reactivity, which may have a certain impact on the research results (Schrimsher and Katie Filtz, 2011). If there are conditions to collect offline data, use the interview form, evaluate from a relatively objective and professional perspective, or provide personalized scale interpretation for subjects after completing the questionnaire, it might improve the probability of subjects to fill in the questionnaire carefully. Thirdly, the current study was only performed in a relatively small sample size of healthy participants, which a lack of diversity in the selection of samples, and future studies may be performed in larger clinical populations to provide more valuable information. Fourthly, the CTQ questionnaire used in this study only emphasizes the variety and frequency of trauma rather than intensity or psychological damage, which can be investigated in further studies. Fifth, no content validity studies for the CTQ-33 have been performed to our knowledge, which may be conducted in the future. Finally, the study only used the total score of CTQ-33 to indicate the level of childhood trauma suffered by an individual that could arise the situation that trauma victims have not experienced trauma in each category of the CTQ-33. Future studies may need to consider different subtypes of trauma.

## Conclusion

In conclusion, in this study, we found that the CTQ-33 has an acceptable factor structure and long-term (over 1 year) test-retest reliability. Meanwhile, we replicated previous findings showing positive relationships between levels of different childhood trauma subtypes in the surveyed adolescents using the CTQ-33. Our findings support that the CTQ-33 is reliable to assess traumatic events in Chinese adolescents over relatively long intervals.

## Data Availability

The raw data supporting the conclusions of this article will be made available by the authors, without undue reservation.

## Footnotes

^1^www.wjx.xn
